# miR-34a Regulates Sperm Motility in Zebrafish

**DOI:** 10.3390/ijms18122676

**Published:** 2017-12-10

**Authors:** Wenjie Guo, Binyue Xie, Shuting Xiong, Xufang Liang, Jian-Fang Gui, Jie Mei

**Affiliations:** 1 College of Fisheries, Key Laboratory of Freshwater Animal Breeding, Ministry of Agriculture, Huazhong Agricultural University, Wuhan 430070, China; guowenjie@mail.hzau.edu.cn (W.G.); heyan@mail.hzau.edu.cn (B.X.); xiongshuting@webmail.hzau.edu.cn (S.X.); xfliang@mail.hzau.edu.cn (X.L.); jfgui@ihb.ac.cn (J.-F.G.); 2 State Key Laboratory of Freshwater Ecology and Biotechnology, Institute of Hydrobiology, Chinese Academy of Sciences, University of the Chinese Academy of Sciences, Wuhan 430072, China

**Keywords:** miR-34a, knockout, sperm motility, *gsk3a*

## Abstract

Increasing attention has been focused on the role of microRNAs in post-transcription regulation during spermatogenesis. Recently, the miR-34 family has been shown to be involved in the spermatogenesis, but the clear function of the miR-34 family in spermatogenesis is still obscure. Here we analyzed the function of miR-34a, a member of the miR-34 family, during spermatogenesis using miR-34a knockout zebrafish generated by the clustered regularly interspaced short palindromic repeats/associated protein 9 (CRISPR/Cas9) system. miR-34a knockout zebrafish showed no obvious defects on testis morphology and sperm quantity. However, we found a significant increase in progressive sperm motility that is one of the pivotal factors influencing in vitro fertilization rates, in the knockout zebrafish. Moreover, breeding experiments showed that, when miR-34a-knockout male zebrafish mated with the wide-type females, they had a higher fertilization rate than did the wide-type males. Glycogen synthase kinase-3a (*gsk3a*), a potential sperm motility regulatory gene was predicted to be targeted by miR-34a, which was further supported by luciferase reporter assays, since a significant decrease of luciferase activity was detected upon ectopic overexpression of miR-34a. Our findings suggest that miR-34a downregulates *gsk3a* by targeting its 3′ untranslated region, and miR-34a/*gsk3a* interaction modulates sperm motility in zebrafish. This study will help in understanding in the role of the miR-34 family during spermatogenesis and will set paths for further studies.

## 1. Introduction

Spermatogenesis is a complicated and highly regulated process in which diploid spermatogonia proliferate and differentiate into mature spermatozoa through three distinct phases: mitosis, meiosis, and spermiogenesis [1,2,3]. Normal spermatogenesis is essential for reproduction and depends upon the regulation of gene expression [4,5,6]. Studies have been performed at molecular and epigenetic levels to elucidate the molecular basis of spermatogenesis and the specific role of these molecules in the function of spermatozoa [7,8,9,10]. Several master genes, such as cyclic AMP-responsive element modulator (*CREM*) [11], sperm-associated antigen 6 (*Spag6*) [12], sperm-associated antigen 6 (*Spag16*) [13], phosphoglycerate kinase 2 (*Pgk2*) [14], have been identified to be involved in spermatogenesis and affect the functions of spermatozoa. Recently, noncoding RNAs (ncRNAs) including microRNAs (miRNAs), piwi-interacting RNAs (piRNAs), and long ncRNAs (lncRNAs) have been considered to have crucial roles in spermatogenesis [15]. These molecules are gradually becoming the focus of studies in the field of gametogenesis.

miRNAs (≈22 nt) are a highly conserved class of ncRNAs, which control gene expression usually via binding to the 3′ untranslated regions (3′UTRs) of many protein-coding transcripts [16]. miRNAs have important roles in mediating various biological functions, including cell division [17], differentiation [18], migration [19], and apoptosis [20]. Recent studies reported that several miRNAs are expressed and exhibit dynamic expression profiles during spermatogenesis [21,22], and may have a role in this process. The role of specific miRNAs such as miR-15a, miR-184, and miR-384 is demonstrated in the process of spermatogenesis. Moreover, miR-15a and its target gene cyclin T2 (*Ccnt2*) associates in early spermatogenesis [23]. Similarly, miR-184 was expressed primarily in testis, which probably was involved in the post-transcription regulation of nuclear receptor corepressor 2 (*Ncor2*) in mammalian spermatogenesis [24]. Although many miRNAs have been detected in testis or spermatozoa, their precise roles and target genes in spermatogenesis or spermatozoa are poorly defined.

Sperm motility is a major factor in judging sperm quality and an important determinant for fertilization success. In recent decades, several sperm motility regulatory genes have been discovered, such as cation channel of sperm (*CatSper*) [25], rab-like 2 (*RABL2*) [26], *gsk3a* [27], and cystic fibrosis transmembrane conductance regulator (*CFTR)* [28]. Recently, it has become evident that miRNAs are involved in regulation of sperm motility. In porcine, a significant increase in the expression of two miRNAs, let-7d and let-7e, was identified in low sperm motility groups compared to a normal group [29]. It has been demonstrated that high expression of miR-27b has a significant relationship with low sperm progressive motility in humans [30]. The miR-34 family is highly evolutionarily conserved in vertebrates and contains three closely related members: miR-34a/b/c [31]. Earlier studies have proved that the miR-34 family acts as regulators of gene expression in cell cycle progression [32], senescence [33], and apoptosis [34]. Other evidences suggest that the miR-34 family also has a crucial role in spermatogenesis and sperm motility [35,36]. At present, however, the precise role of the miR-34 family during spermatogenesis is still poorly understood in vivo. In this study, we started with miR-34a to explore the functional roles of the miR-34 family in spermatogenesis using miR-34a homozygous knockout zebrafish produced by the CRISPR/Cas9 system, and tried to identify the targeted gene of miR-34a using the bioinformatics and luciferase reporter assay.

## 2. Results

### 2.1. Expression of miR-34a during Zebrafish Testis Development

**Figure 1:**
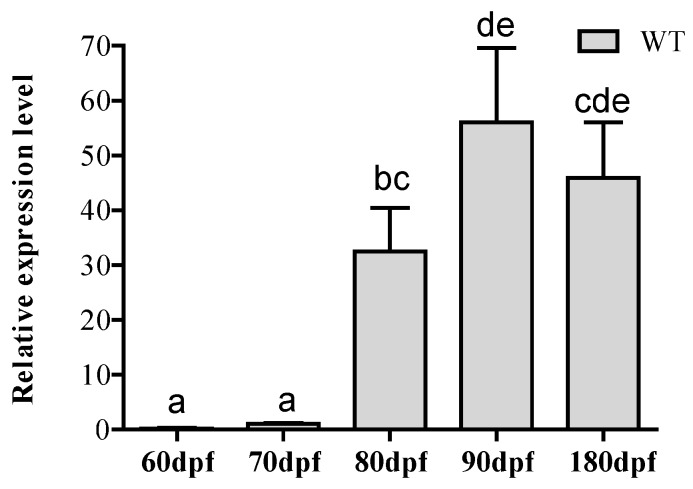
Relative expression levels of miR-34a in the testis of wild-type (WT) zebrafish at different developmental stages.

To investigate the expression pattern of miR-34a in zebrafish testis, its expression was examined at different developmental stages of testis including 60, 70, 80, 90, 180 days post-fertilization (dpf). As shown in Figure 1, the expression of miR-34a was relatively low at 60 dpf, gradually increased as testis developed, and reached the maximum at 90 dpf (mature testis). However, its expression decreased at 180 dpf in aging testis. This varying expression of miR-34a made us speculate that miR-34a might play a role in testis development.

### 2.2. Establishment of miR-34a Knockout Zebrafish Model

**Figure 2:**
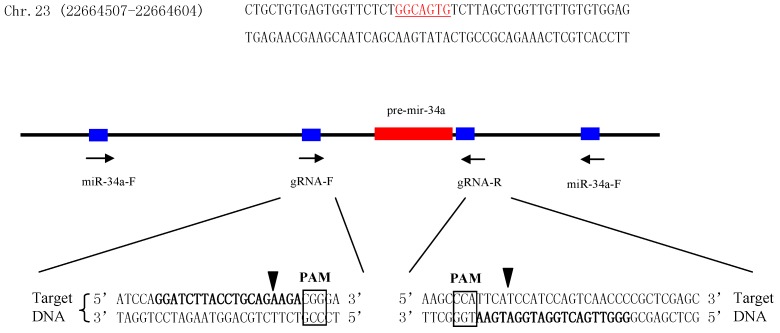
Schematic representation of the CRISPR/Cas9 strategy for miR-34a deletion and mutation detection. The pre-miR-34a sequence was shown in bold, and the seed sequence is underlined. Left arrows indicate that the sequences were consistent with the given sequences; right arrows indicate that the sequences were reverse-complement of the given sequences. The box indicates the PAM sequence.

**Figure 3:**
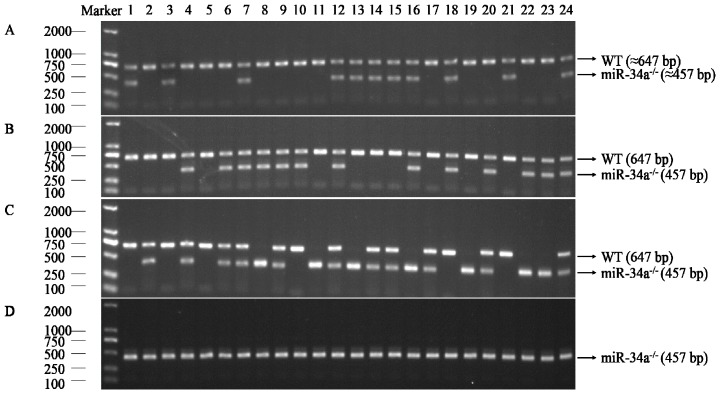
Establishment of miR-34a knockout zebrafish model. (**A**) Lanes 1–24: PCR products from F0 generation individuals; (**B**) Lanes 1–24: PCR products from F1 generation individuals; (**C**) Lanes 1–24: PCR products from F2 generation individuals; (**D**) Lanes 1–24: PCR products from F3 generation individuals.

To further explore the role of miR-34a during testis development, miR-34a knockout zebrafish was generated using a CRISPR strategy. Firstly, two guide RNAs (gRNAs) were designed to target the upstream and downstream regions of pre-miR-34a, respectively (Figure 2). Next, both gRNAs and Cas9 mRNA were co-injected into 1- to 2-cell stage zebrafish embryos. The target region was amplified by the polymerase chain reaction (PCR), and the amplification products were sequenced. DNA sequencing results indicated that gRNAs used in the present study could induce Cas9 cutting activity in the target sites. When the injected embryos grew to the adult stage, 24 individuals were screened, and 11 individuals had the potential to be used as F0 mutants (Figure 3A). An F0 mutant with high deletion was crossed with a wild type to obtain an F1 heterozygous fish. Of 24 F1 adults, 13 fish were confirmed as heterozygote (Figure 3B). The male and female F1 mutant alleles were mated to generate F2 generation. As expected, the percentage of homozygous null allele mutants in F2 offspring was almost 25 (7/24, Figure 3C). These homozygous mutants were mated with each other to produce 100% F3 homozygous null mutants (24/24, Figure 3D) for subsequent study.

### 2.3. Effects of miR-34a Knockout on Zebrafish Sperm Motility

**Figure 4:**
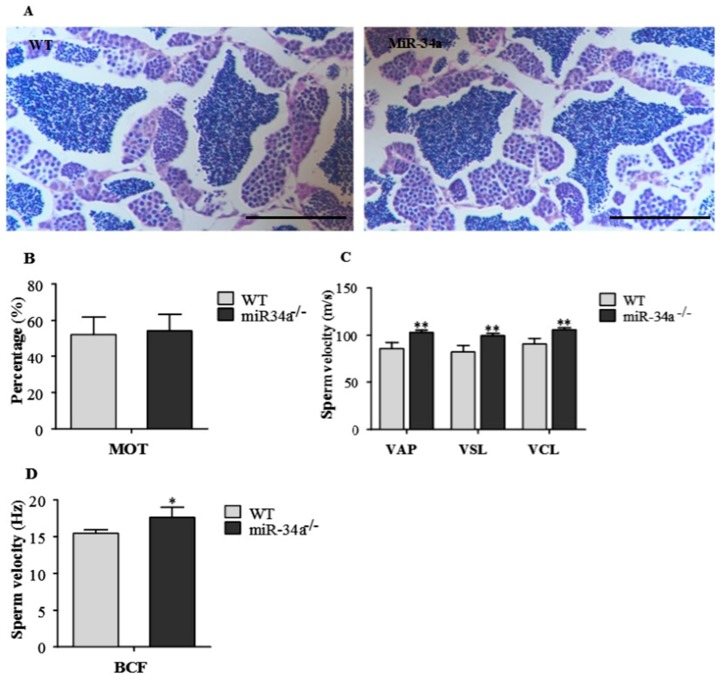
Testis morphology and sperm motility parameters between WT and miR-34a^−/−^ zebrafish. (**A**) H&E staining of testicular tissue. Scale bar: 100 μm; (**B**) Percentages of motile sperm for WT and miR-34a^−/−^ zebrafish; (**C**) Sperm velocity parameters of progressive sperm for WT and miR-34a^−/−^ zebrafish; (**D**) BCF of progressive sperm for WT and miR-34a^−/−^ zebrafish. * *p* < 0.05 and ** *p* < 0.01.

We analyzed the phenotype of miR-34a knockout zebrafish focusing on spermatogenesis and spermatozoa activity. The morphology of the testis was similar between WT and miR-34a^−/−^ zebrafish (Figure 4A), suggesting that disruption of miR-34a did not affect the testis morphology. In addition, miR-34a knockout zebrafish can be mated to generate viable offspring, which indicated that miR-34a deletion does not affect normal spermatogenesis. Therefore, we tried to check the quantity and quality of spermatozoa. No significant difference in sperm quantity was detected between wild-type and miR-34a knockout zebrafish. Moreover, there was no significant difference in the percentage of motile sperm (MOT) between wild-type and miR-34a knockout zebrafish (Figure 4B). However, the values of several progressive sperm motility parameters (VAP: average pathway velocity; VSL: straight-line velocity; VCL: curvilinear velocity; BCF: beating cross frequency) significantly increased in miR-34a^−/−^ zebrafish compared to the WT zebrafish (Figure 4C,D), indicating that sperm from miR-34a^−/−^ zebrafish, when they mate with wild-type eggs, might improve the fertilization rate.

**Figure 5:**
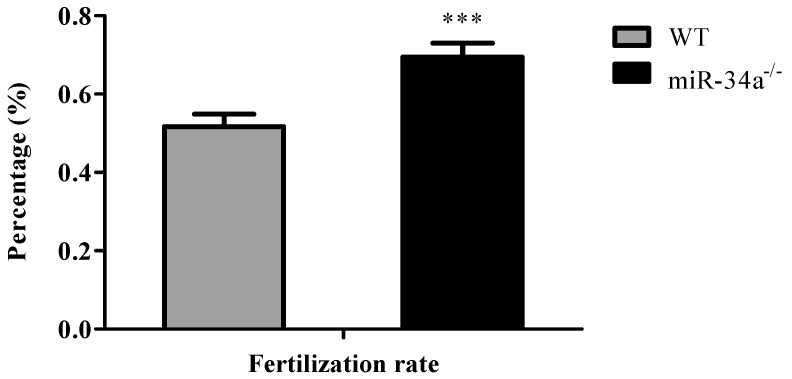
Fertilization rates of WT and miR-34a^−/−^ male zebrafish when they mated with WT female zebrafish. *** *p* < 0.001.

To test the fertilization rate, breeding experiments were carried out with male miR-34a^−/−^ zebrafish vs. female wide-type zebrafish and male wide-type zebrafish vs. female wide-type zebrafish. The result of breeding experiments showed that, when miR-34a^−/−^ zebrafish mated with eggs from wide-type zebrafish, miR-34a^−/−^ zebrafish had a higher fertilization rate than the wide-type zebrafish (Figure 5).

### 2.4. Potential Target Genes of miR-34a

**Figure 6:**
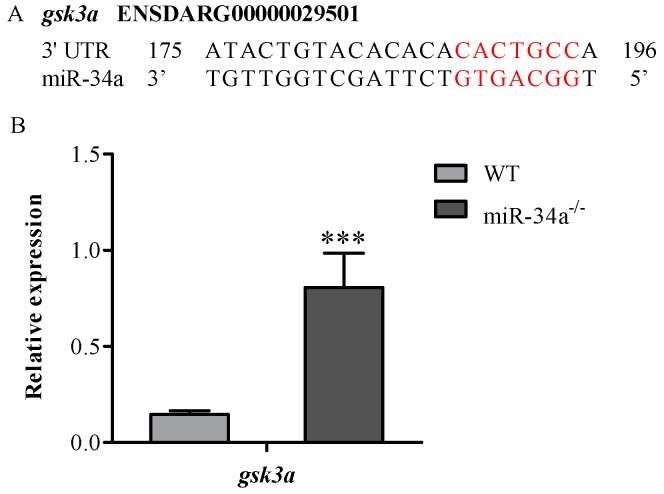
Knockout of miR-34a affected the expression levels of *gsk3a* in zebrafish testis. (**A**) The alignment of mature miR-34a with *gsk3a* 3′UTR. Red indicates miR-34a seed sequence and the putative miR-34a binding site in *gsk3a*; (**B**) The fold changes of the expression of *gsk3a* mRNA in the testis of WT and miR-34a^−/−^ zebrafish examined by RT-PCR. *** *p* < 0.001.

Using the Targetscan bioinformatics algorithm, we found that *gsk3a* was a putative target gene of miR-34a, since there was a binding site for miR-34a in the 3′UTR of *gsk3a* (Figure 6A). Moreover, qRT-PCR analysis revealed that *gsk3a* mRNA expression was upregulated in the testis of miR-34a^−/−^ zebrafish compared with WT zebrafish (Figure 6B), suggesting that *gsk3a* is a candidate miR-34a target gene for affecting sperm motility.

### 2.5. Luciferase Reporter Assays

**Figure 7:**
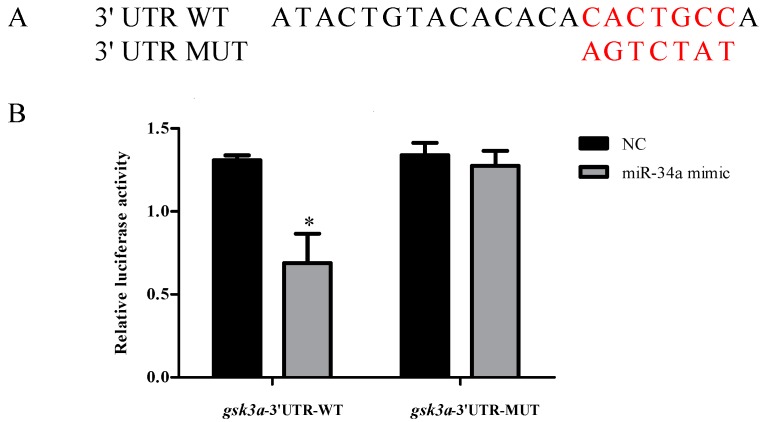
miR-34a directly targets *gsk3a*. (**A**) The sequence information of the putative *gsk3a* 3′UTR binding site in wild type (UTR WT) and mutant (UTR MUT); (**B**) Luciferase assays of miR-34a co-transfection with UTR WT plasmid and UTR MUT plasmid in HEK293T cells. The luciferase activity of WT plasmid was reduced by miR-34a. NC stands for negative control mimic. Error bars indicate mean ± SD, *n* = 3. Student’s *t*-test was used for statistical analysis (* *p* < 0.05).

To determine whether zebrafish miR-34a directly targets *gsk3a*, luciferase reporter experiments were carried out by inserting the 3′UTR of *gsk3a* into the C-terminus of Firefly luciferase in the pmirGLO reporter vector. The binding site of miR-34a (CACTGCC) in the constructed wild-type plasmid was replaced with AGTCTAT by site-directed mutagenesis (Figure 7A). As expected, the relative luciferase activity was repressed in the wild-type zebrafish construct, whereas the repression was abrogated when the mutant construct was used (Figure 7B).

## 3. Discussion

miRNAs were first discovered in *C. elegans* as early as the 1990s [37], but the biological functions have not attracted considerable attention until a number of endogenous miRNAs were identified in worms, flies, and mammals. To date, miRNAs have been shown to have important regulatory roles in a variety of biological processes, including stem cell differentiation [38], signaling transduction [39], developmental regulation [40] and in diseases [41,42]. In vertebrates, the miR-34 family is an evolutionary conserved family, including three members: miR-34a, miR-34b, and miR-34c. The miR-34 family was usually recognized as the tumor suppressors that control cell proliferation and cell-cycle progression through silencing oncogenic targets [43]. Recent studies revealed the miR-34 family might have roles in spermatogenesis and sperm motility [35,36]. However, its precise roles in spermatogenesis remain unclear. Therefore, zebrafish was used as a model in the current study to observe the functions of miR-34a is in spermatogenesis and sperm motility.

The CRISPR/Cas9 system is an ideal gene knockout tool to characterize gene function in vivo [44]. In the present study, the CRISPR/Cas9 was used to generate miR-34a knockout zebrafish (Figure 2), with the mutagenesis efficiency more than 45% (Figure 3A). Our results showed that testis morphology in miR-34a^−/−^ zebrafish was normal, but the sperm motility was increased when compared with the WT, indicating that deletion of miR-34a leads to an enhancement of sperm motility in zebrafish. Previously, it has been reported that cell motility could be regulated by miR-34a. miR-34a functioned as a tumor suppressor gene to inhibit uveal melanoma cell motility through the downregulation of c-Met [45]. When the endogenous miR-34a was knocked down using short interfering RNA (siRNA), Wharton’s jelly MSCs (WJ-MSCs) motility was increased [46]. These studies suggested that there is a negative correlation between miR-34a and cell motility.

*gsk3a* has been confirmed to be essential for normal sperm motility, and loss of *gsk3a* led to male infertility due to the decrease in sperm motility [27]. Interestingly, a binding site of miR-34a was found in 3′UTR of zebrafish *gsk3a*. MicroRNAs usually regulate gene expression by posttranscriptional repression and degradation of target mRNA [47,48,49]. *gsk3a* expression in miR-34a^−/−^ zebrafish was about 5.5 times that in wild-type zebrafish. The luciferase reporter assay further indicated that *gsk3a* gene is a target gene of miR-34a in zebrafish. Adenosine triphosphate (ATP) is essential for sperm motility, which is obtained mainly from two metabolic pathways: mitochondrial oxidative phosphorylation and glycolysis [50]. It has been revealed that miR-34a may be involved in mitochondrial oxidative phosphorylation and regulate ATP content. For example, in HepG2 cells, miR-34a reduced ATP contents by targeting ATP synthase subunit 5s (ATP5S), which is a subunit of the key enzyme of oxidative phosphorylation and is responsible for ATP production in mammals [51]. In circulating endothelial cells, overexpression of miR-34a could impair mitochondrial oxidative phosphorylation and reduced ATP production by targeting cytochrome c [52]. gsk3, a serine-threonine protein kinase, can phosphorylate and inactivate glycogen synthase [53], which may lead to decrease glycogen synthesis, and more glucose can probably be supplied for glycolysis to generate more ATPs. In *gsk3a* ^−/−^ mice, it was noted that sperm motility parameters were impaired and ATP levels were reduced, suggesting an association between *gsk3a* and glycolytic metabolism [27]. In conclusion, miR-34a may regulate sperm motility by targeting *gsk3a* in zebrafish.

## 4. Materials and Methods

### 4.1. Zebrafish Strain

Wild-type AB zebrafish strain used in this study was acquired from the Institute of Hydrobiology, Chinese Academy of Science (Wuhan, China). All experiments procedures involving zebrafish were approved by the institution animal care and use committee of Huazhong Agricultural University.

### 4.2. Design of CRISPR/Cas9 Target Site and Single Guide RNA (sgRNA) Synthesis

CRISPR/Cas9 target sites were designed as previously described [54], the sequences of which were listed in Table 1 and Figure 1. Each synthetic-guide RNA (sgRNA) was synthesized by inserting Cas9 target site sequence between the T7 promoter and sgRNA scaffold sequence of pMD19-gRNA scaffold plasmid followed by in vitro transcription.

### 4.3. Capped mRNA Synthesis

Cas9 expression plasmid (pCS2-nCas9n, Addgene plasmid # 47929) was linearized by XbaI and capped Cas9 mRNA was produced via T7 in vitro Transcription Kit (Life Technologies, Gaithersburg, MD, USA). The concentration of the capped mRNA was assessed with NanoDrop (Thermo Scientific, Waltham, MA, USA) and the quality was examined by agarose gel electrophoresis.

### 4.4. Microinjection, Mutation Analyses, and Mutant Lines Establishment

Embryos (*n* > 200) at the 1- to 2-cell stage were microinjected with 300 pg Cas9 mRNA and 20 pg each sgRNA. Uninjected embryos were used as controls. All embryos were kept at constant water temperature (28.5 °C) on a 14:10 h light–dark cycle. Genomic DNA was isolated from 20 normally developing wild-type or microinjected embryos at 2 dpf. The mutations of targeted genomic regions were determined by polymerase chain reaction (PCR). The PCR primers used are displayed in Table 1. The putative F0 founder fish were reared to sexual maturity, and 24 adult fish were randomly selected for mutation examination by PCR. Two individuals containing high deletion were outcrossed with wild-type fish to produce F1 generation. F1 heterozygous fish having the same mutant were crossed to obtain F2 zebrafish, from which the homozygous mutants were screened out. Homozygous mutant genotypes of F3 offspring were also checked by PCR and used for subsequent analysis.

### 4.5. Evaluation of Sperm Motility and Fertilization Rate

Sperm samples of the same age from both the control and the F3 generation homozygous fish were kept under the same culture conditions and stored in preservative fluid (63 mM NaCl, 19 mM KCl, 1.3 mM CaCl_2_, 4.7 mM MgSO_4_·7H_2_O, 2.5 mM NaHCO_3_, pH 7.4). Parameters of sperm motility were determined by a computer-assisted sperm analysis (CASA) system (Hamilton-Thorne Biosciences, Beverly, MA, USA) [55]. Immediately after activation in room temperature (25 ± 1 °C), sperm motility was evaluated under 4× magnifications after adjusting the number of sperm from 200 to 300 in a microscope field. Sperm in each sample was evaluated in quadruplicate. Motility parameters included the percentage of MOT (%), VAP (μm/s), VSL (μm/s), VCL (μm/s), and BCF (%).

To compare the fertilization rate, miR-34a^−/−^ and wide-type male zebrafish were mated with the wide-type females adopting natural spawning, respectively. All parental fish were produced through artificial reproduction almost at the same time and cultured under the same circulating water system and conditions. Each breeding experiment contained 6 male and 6 female fish and was performed in triplicate. The fertilization rate was determined as the number of development eggs in relation to the total eggs. Four hundred eggs obtained from each breeding experiment were tested for evaluation of fertilization rate.

### 4.6. RNA Extraction and Quantitative Real-Time PCR (qRT-PCR) Analysis

Total RNA was extracted from testes using the Qiagen miRNeasy mini Kit (Qiagen, Valencia, CA, USA) according to the manufacturer’s instructions. Extracted RNA was reverse transcribed into cDNA using GoScript™. Reverse Transcription System (Promega, Madison, WI, USA). Real-time reverse transcription PCR (qRT-PCR) was performed in triplicate on Bio-Rad CFX96^TM^ (Bio-Rad, Hercules, CA, USA) Real-Time PCR system using a standard IQTM SYBR Green Supermix Kit (Bio-Rad Laboratories, Hercules, CA, USA). For miRNA and mRNA RT-PCR, *U6* and *β-actin* were used as endogenous control, and data were analyzed using the comparative 2^−ΔΔ*C*t^ method. The primers for PCR amplification are presented in Table 2.

### 4.7. Luciferase Reporter Assay

Using the Targetscan bioinformatics algorithm, a putative target site for miR-34a was detected in the 3′UTR of *gsk3a*, which was cloned and inserted into the pmir-GLO plasmid (Promega, Madison, WI, USA). The target site sequence (CACTGCC) in the constructed wild-type plasmid was replaced with AGTCTAT through site-directed mutagenesis, as described previously [56]. HEK-293 T cells seeded in 24-well plates were co-transfected with plasmid (25 ng wild-type or mutant) and miRNA (50 nM mimics or negative control) using DharmaFECT transfection reagent (Dharmacon, Lafayette, CO, USA). Luciferase activity was assessed at 24 h after transfection using a Dual Luciferase reporter assay system (Promega, Madison, WI, USA) as previously described [57]. Relative reporter activities were determined by normalizing Firefly activity to Renilla activity. All transfection experiments were performed in triplicate.

### 4.8. Statistical Analysis

Data are presented as mean ± SD, and the differences between groups were analyzed using a Student’s *t*-test. A *p*-value less than 0.05 was considered statistically significant.

## Figures and Tables

**Table 1:** miR-34a knockout target sites and primer for mutation detection.

Items	Sequences (5′–3′)	PAM
gRNA-F	GGATCTTACCTGCAGAAGAC	GGG
gRNA-R	GGGTTGACTGGATGGATGAA	TGG
g-miR-34a-F	GGACTTGTGACTGCTGTAATTCC	
g-miR-34a-R	CTAATGAAATGACTCAGGCTAC	

**Table 2:** The primers used for conducting qRT-PCR analysis.

Primers	Sequences (5′-3′)	Size of the Products (bp)
miR-203a stem loop	CTCAACTGGTGTCGTGGAGTCGGCAATTCAGTTGAG	
*miR-34a*-F	GCGTGGCAGTGTCTTAGCTG	57
*miR-34a*-R	ACTGGTGTCGTGGAGTCGGC	
*U6*-F	TGCTCGCTACGGTGGCACA	111
*U6*-R	AAAACAGCAATATGGAGCGC	
*gsk3a*-F	TAAAGGGGCACAAGAGGTTC	187
*gsk3a*-R	TGTCGCTGATAGATATTTCGTC	
*β-actin*-F	CGAGCAGGAGATGGGAACC	102
*β-actin*-R	CAACGGAAACGCTCATTGC	
